# Effects of storage time and temperature on greenhouse gas samples in Exetainer vials with chlorobutyl septa caps

**DOI:** 10.1016/j.mex.2018.06.016

**Published:** 2018-06-28

**Authors:** Derek R. Faust, Mark A. Liebig

**Affiliations:** Northern Great Plains Research Laboratory, USDA-Agricultural Research Service, P.O. Box 459, Mandan, ND, 58554, USA

**Keywords:** Storage of greenhouse gas samples, Certified gas standards, Septa-capped vials, Static chamber method, Storage recommendations

## Abstract

Measurement of greenhouse gas (GHG) flux using static chamber methods typically occurs immediately following sample collection. However, situations may arise requiring sample storage prior to analysis by gas chromatography. The objective of this study was to determine effects of storage time and temperature on carbon dioxide (CO_2_), methane (CH_4_), and nitrous oxide (N_2_O) concentrations in vials containing “low” and “high” concentrations of certified standards. Samples were stored for 3, 7, 14, 28, and 84 days at four storage temperatures: room temperature, 25 °C, 4 °C, and −10 °C. Results indicated low and high concentration standards were not impacted by sample storage up to 28 days at any storage temperature. After 84 days, CO_2_ concentrations were 0.6–14.4% lower than expected while CH_4_ concentrations were up to 22% greater than expected. Results from future studies will allow for further refinement of scientifically supported guidance regarding appropriate storage temperature and time of GHG samples.

• Few studies have examined impacts of storage time and temperature on GHG samples retained in traditional septa-capped vials.
• Effects of storage time and temperature on GHG samples were examined.
• Based on this study, GHG samples can be stored for up to 28 days at temperatures ranging from −10 °C to 25 °C.

## Graphical abstract

**Figure:**
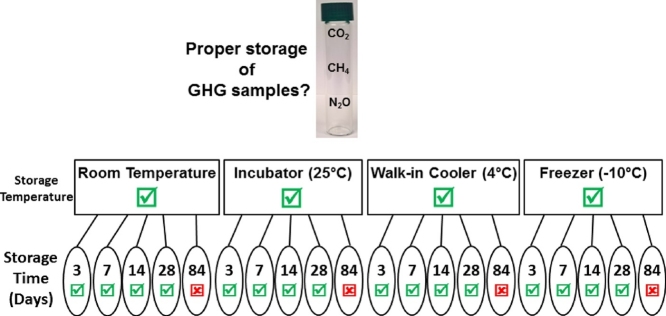

**Specification Table**

**Table:** 

Subject Area	*Environmental Science*
More specific subject area:	*Biogeochemistry*
Method name:	*Storage of greenhouse gas samples*
Name and reference of original method	*A.R. Mosier, G.L. Hutchinson, Nitrous oxide emissions from cropped fields. J. Environ. Qual. 10 (1981)169-173.* *T.B. Parkin, R.T. Venterea, Chamber-based trace gas flux measurements, in: R.F. Follet (Ed.), GRACEnet Sampling Protocols, 2010. P. 3-1 to 3-39. Available at https://www.ars.usda.gov/anrds/gracenet/ (verified 8 May 2018).* *C. de Klein, M. Harvey (Eds.), Nitrous Oxide Chamber Methodology Guidelines. Version 1.1, 2015. Available at: www.globalresearchalliance.org (verified 8 May 2018).*
Resource availability	*https://www.ars.usda.gov/anrds/gracenet/*

## Method details

Measurement of greenhouse gas (GHG) flux occurs in a variety of ecosystems and landscapes, often with the goal of assessing carbon (C) and nitrogen (N) dynamics and associated ecosystem contributions to global warming potential. The static chamber method for collection of GHG samples is one of the most commonly used methods for determining GHG flux from agricultural soils over the past 30 years and requires samples be stored in a vessel before analysis [1]. In many cases, analysis of samples begins as soon as possible after collection [2,3]. However, situations may arise whereby analysis is delayed and samples must be stored (e.g., time lag due to transport of samples from field to laboratory; gas chromatograph immediately unavailable due to sample analysis backlog; gas chromatograph or autosampler is inoperable for maintenance, servicing, or troubleshooting). Studies and associated guidance can be found that established Exetainer® vials with chlorobutyl septa (Labco Limited, Lampeter, UK) as the recommended vessel for GHG storage, along with other associated vial and sampling recommendations [[2], [3], [4], [5], [6], [7]]. Unfortunately, guidance is lacking regarding appropriate storage temperature and time for GHG samples retained in septa-capped vials.

Segschneider et al. [4] observed only butyl rubber septa provided an effective seal for up to seven days among butyl, ethylene propylene diene monomer rubber, silicone, and Teflon faced butyl stoppers and septa. Duration of sample storage for less than 15 days in glass vials was ranked as “very good” by Rochette and Eriksen-Hamel [7] based on contamination and leakage potential. Some studies evaluated storage of gas samples using the amount of time vials maintained evacuation volume. Rochette and Bertrand [5] observed vials with double-wadded septa could be evacuated up to 63 days prior to use and evacuated Exetainer vials maintained evacuation volume well for 135 days. Use of both butyl rubber and silicone septa decreased the loss of vacuum with time (from 11% with only the rubber septa to 2% with both) and provided a more reliable seal [5]. Parkin and Venterea [2] found screw cap vials with butyl rubber septa held pressure better after 3 and 13 days compared to crimp top serum vials.

Most studies examining impacts of storage time on GHG samples focused on nitrous oxide (N_2_O), leaving information gaps regarding storage of carbon dioxide (CO_2_) and methane (CH_4_). No significant leakage for C isotope value was detected in Exetainer vials for up to 14 days [8]. Laughlin and Stevens [9] tested 12-mL Exetainer vials monthly for one year of storage of ^15^N-labeled N_2_O and N_2_. Concentration of N_2_O did not decrease significantly for up to 8 weeks, but had decreased by 34% after a year of storage at room temperature in Exetainer vials [9]. Samples with N_2_O concentration of 10 μL L^−1^ and storage periods of 14 and 126 days had between 92 and 98% retention from vials using butyl rubber and double-wadded septa, respectively [5]. A butyl rubber septa-sealed vial with N_2_O concentration of 1 μL L^−1^ retained 90% of the gas for storage period of 365 days [3]. Another suggested alternative for accurate N_2_O concentration determination was storage and analysis of standards along with samples [9]. Only one study, evaluated the effects of storage temperature, observing storage at 5 and 20 °C had no effect on N_2_O concentration in Exetainer vials [9].

Due to the lack of studies documenting effects of storage time and temperature on GHG samples available in peer-reviewed literature or guidance documents, the objective of this study was to determine effects of storage time and temperature on carbon dioxide (CO_2_), methane (CH_4_), and nitrous oxide (N_2_O) in septa-capped vials. This will be the first study to systematically evaluate a broad range of both storage temperatures and times, will improve reliability of GHG values reported in the literature, and provide guidance for appropriate storage temperature and time for GHG samples. We hypothesized colder storage temperatures (walk-in cooler and freezer) would provide greater storage stability of GHG samples than at room temperature or 25 °C (incubator) and storage of GHG samples longer than 28 days would result in unacceptable amounts of leakage of measured gases.

## Method validation

### Sample preparation and storage

**Fig. 1:**
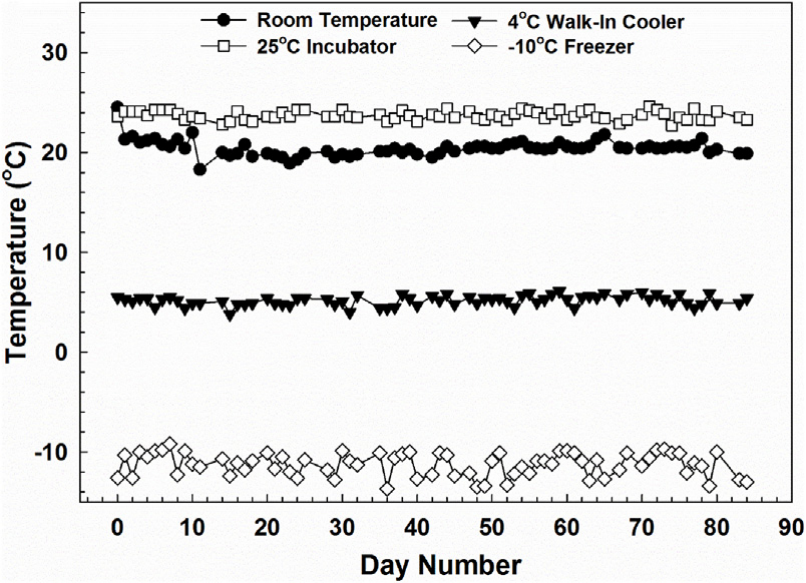
Storage temperatures over the 84 day study. Storage locations were laboratory workbench at room temperature, incubator set to 25 °C, walk-in cooler set to 4 °C, and freezer at −10 °C. Temperature measurements were obtained using an Omega SC-GG-K-30-36-PP thermocouple (Omega Engineering, Inc., Stamford, CT) positioned in each location using a VWR traceable dual laser infrared thermometer (VWR International LLC, Radnor, PA).

Four storage locations differing in ambient temperature were used in the study: laboratory workbench at ambient room temperature, incubator set to 25 °C, walk-in cooler set to 4 °C, and freezer at −10 °C. Storage times were 3, 7, 14, 28, and 84 days. To reduce effects of light, vials for each storage temperature were placed in corrugated cardboard boxes. An Omega SC-GG-K-30-36-PP thermocouple (Omega Engineering, Inc., Stamford, CT) was positioned in each box such that storage temperature measurements could be monitored periodically using a VWR traceable dual laser infrared thermometer (VWR International LLC, Radnor, PA). Mean ± standard deviation temperatures over the 84 day study were 20.4 ± 0.8 °C for room temperature, 23.5 ± 0.4 °C for incubator, 5.2 ± 0.5 °C for walk-in cooler, and −11.3 ± 1.2 °C for freezer (Fig. 1).

Before placement in their respective storage locations, Exetainer® vials sealed with chlorobutyl rubber septa (Labco Limited, Lampeter, UK) were prepared by purging with ultrapure helium (Praxair Distribution, Inc., Danbury, CT) for 10 s and evacuated for 15 s. After preparation, one set of vials were injected with 15 mL of “low” concentration CO_2_, CH_4_, and N_2_O blended standard and another set of vials were similarly injected with a “high” concentration blended standard. Standards were certified by the National Oceanic and Atmospheric Administration Global Monitoring Division (Boulder, CO) on 22 June 2015 with analysis certificate numbers CB11245-A and CB11061-A for “low” and “high” standards, respectively. Concentrations in the low standard were 380 μL L^−1^ CO_2_, 1.790 μL L^−1^ CH_4_, and 313 nL L^−1^ N_2_O, while concentrations in the high standard were 1000 μL L^−1^ CO_2_, 2.500 μL L^−1^ CH_4_, and 360 nL L^−1^ N_2_O.

For each storage temperature and time treatment combination, four replicate vials of low and high standards were prepared. After the appropriate storage time had passed, vials were placed on a laboratory workbench and allowed to set at room temperature for 30 min before analysis. At this time, vial temperatures had equilibrated to approximately 21.0 °C as measured by a VWR traceable dual laser infrared thermometer (VWR International LLC, Radnor, PA).

### Sample analysis

Concentrations of CO_2_, CH_4_, and N_2_O were determined using gas chromatography on a Varian CP-3800 Gas Chromatograph with CombiPAL autosampler and Varian Star Workstation Version 6.41 software (Agilent Technologies, Santa Clara, CA). Samples were injected and split into two sample loops. One sample loop used ultra-high purity helium (Praxair Distribution, Inc., Danbury, CT) to carry 1 mL of sample through a column and to a thermal conductivity detector for CO_2_ and flame ionization detector for CH_4_. Hydrocarbon-free air and ultra-high purity hydrogen (Praxair Distribution, Inc) were used for combustion in the flame ionization detector. The second sample loop used ultra-high purity helium and a 5% methane, 95% argon blend (Praxair Distribution, Inc., Danbury, CT) to carry 0.5 mL of sample through a column to a ^63^Ni electron capture detector for N_2_O. The gas chromatograph was calibrated with three standard blends of CO_2_ (350, 424, 1998 μL L^−1^), CH_4_ (1.1, 2.08, 10 μL L^−1^), and N_2_O (82, 411, 2018 nL L^−1^) balanced in N_2_ from Scott Specialty Gases (Plumsteadville, PA). Coefficient of Determination (R^2^) values of calibration curves for each analyte (CO_2_, CH_4_, and N_2_O) and every run of samples was ≥0.999.

For quality assurance and quality control purposes, each run of samples included hydrocarbon-free air checks every ten samples (n = 5 for each run). Mean (±standard deviation) percent recoveries of expected concentrations for these quality control air checks were 94.4 ± 4.8% for CO_2_, 103 ± 7% for CH_4_, and 95.3 ± 8.1% for N_2_O. Furthermore, six vials each of the low and high standards used in the study were filled the day of each run. Mean (±standard deviation) percent recoveries of expected concentrations for the low standard were 99.3 ± 4.8% for CO_2_, 98.7 ± 6.0% for CH_4_, and 109.9 ± 3.4% for N_2_O. Mean (±standard deviation) percent recoveries of expected concentrations for the high standard were 90.7 ± 6.0% for CO_2_, 97.9 ± 2.1% for CH_4_, and 99.6 ± 2.5% for N_2_O.

### Statistical analyses

All statistical analyses were conducted in R 3.3.1 [10] using an alpha level of 0.05 to indicate statistical significance. Analysis of variance (ANOVA) was used to test for statistical differences between treatments. Fixed effects were storage temperature, time, and storage temperature x time interaction. Tukey honest significant difference (HSD) post-hoc tests were used if ANOVA tests indicated treatment effects. Normality and variance homogeneity assumptions were met as indicated by Shapiro-Wilk and Bartlett’s tests, respectively. ANOVAs and Tukey HSD tests were conducted individually for CO_2_, CH_4_, and N_2_O concentrations for the low and high standards.

### Results

**Fig. 2:**
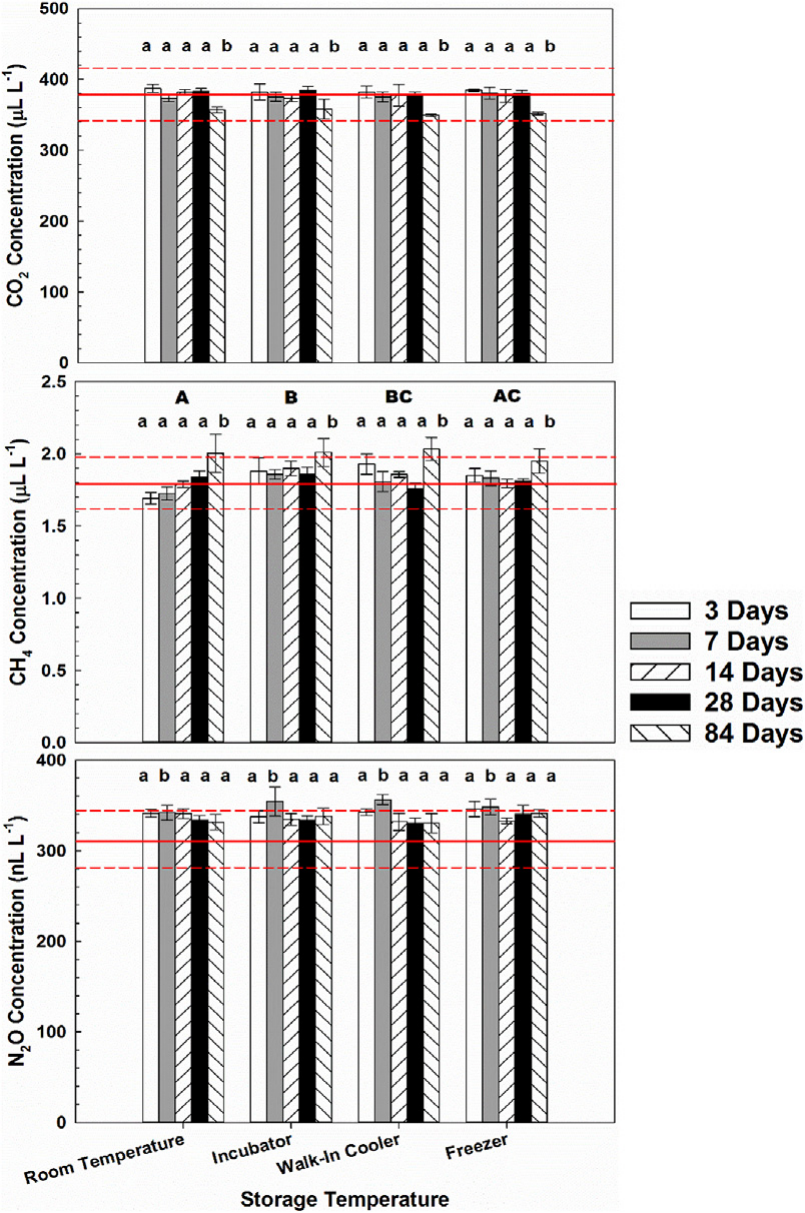
Carbon dioxide (CO_2_), methane (CH_4_), and nitrous oxide (N_2_O) concentrations of a National Oceanic and Atmospheric Administration Global Monitoring Division certified “low” blended standard for different storage temperatures (room temperature, incubator set to 25 °C, walk-in cooler set to 4 °C, and freezer at 10 °C) and storage times (3, 7, 14, 28, and 84 days). Significant differences between storage temperatures and storage times are denoted by different uppercase letters and different lowercase letters, respectively. Solid red lines represent certified standard concentrations, while dotted lines represent ±5% of certified concentrations.

**Fig. 3:**
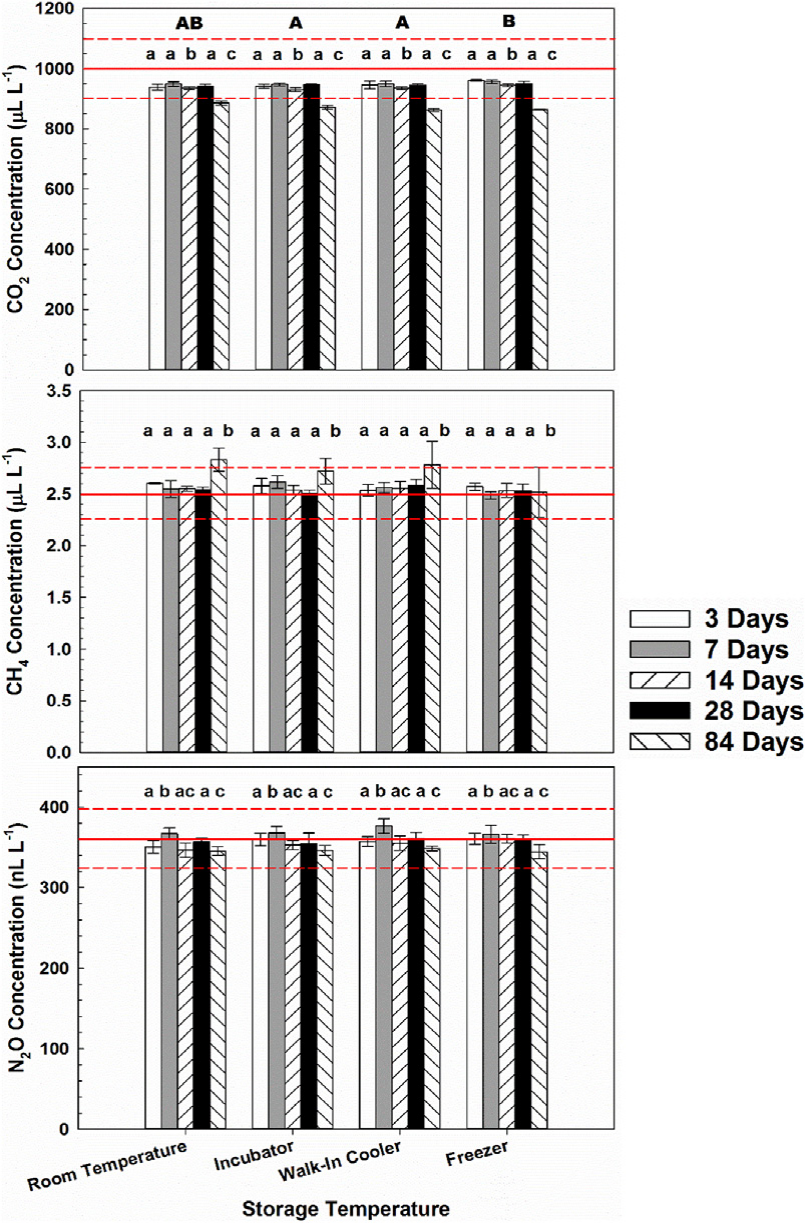
Carbon dioxide (CO_2_), methane (CH_4_), and nitrous oxide (N_2_O) concentrations of a National Oceanic and Atmospheric Administration Global Monitoring Division certified “high” blended standard for different storage temperatures (room temperature, incubator set to 25 °C, walk-in cooler set to 4 °C, and freezer at 10 °C) and storage times (3, 7, 14, 28, and 84 days). Significant differences between storage temperatures and storage times are denoted by different uppercase letters and different lowercase letters, respectively. Solid red lines represent certified standard concentrations, while dotted lines represent ±5% of certified concentrations.

Storage temperature significantly affected CH_4_ concentrations of the low standard (F_3, 57_ = 8.58; P < 0.001) (Fig. 2) and CO_2_ concentrations of the high standard (F_3, 60_ = 6.11; P = 0.001) (Fig. 3). Similarly, a significant storage temperature x storage time interaction was observed for CH_4_ low standard concentrations (F_12, 57_ = 3.03; P = 0.002) and CO_2_ high standard concentrations (F_12, 60_ = 5.04; P < 0.001). Conversely, storage time significantly influenced CO_2_, CH_4_, and N_2_O concentrations for both low and high standards (All P < 0.001) (Fig. 2, Fig. 3). This time effect primarily resulted from significant differences between storage for 84 days and all other storage times. Concentrations of GHGs measured were generally within 5% of certified concentrations regardless of storage temperature and time with one exception-N_2_O concentrations of the low standard were consistently 30–40 nL L^−1^ greater than the certified concentration of 313 nL L^−1^ (Fig. 2).

### Brief discussion

Due to the importance of obtaining accurate and precise concentration data and extensive use of static chamber methodology for calculation off GHG fluxes [1], this study examined effects of storage temperature and time on CO_2_, CH_4_, and N_2_O concentrations in septa-capped vials. Significant effects of both storage time and temperature were observed. Although significant differences by storage temperature were observed for CH_4_ low standard and CO_2_ high standard, differences were small and likely the result of statistical power to detect differences between treatments and interaction effects between storage time and temperature. The interactive effect of time was apparent in both circumstances such that 84 days of storage resulted in consistently higher than expected CH_4_ low concentrations and lower than expected CO_2_ high concentrations (Fig. 2, Fig. 3). Other possible mechanisms could include leakage or contamination of GHG samples, both of which increase with storage duration [5].

In support of the hypothesis, storage time affected concentrations of CO_2_, CH_4_, and N_2_O in both low and high standards, with the most apparent effect after 84 days of storage (Fig. 2, Fig. 3). On average, CO_2_ concentrations for both low and high standards after 84 days were less than concentrations for other storage times and 5% and 10% lower than certified concentrations of the standards, respectively. Concentrations of CH_4_ in both low and high standards were generally greater than concentrations for other storage times and at least 10% greater than the certified concentrations. Concentrations of CH_4_ greater than certified concentrations in low and high standards are difficult to explain, but could be due to sample contamination over the course of 84 days of storage.

**Fig. 4:**
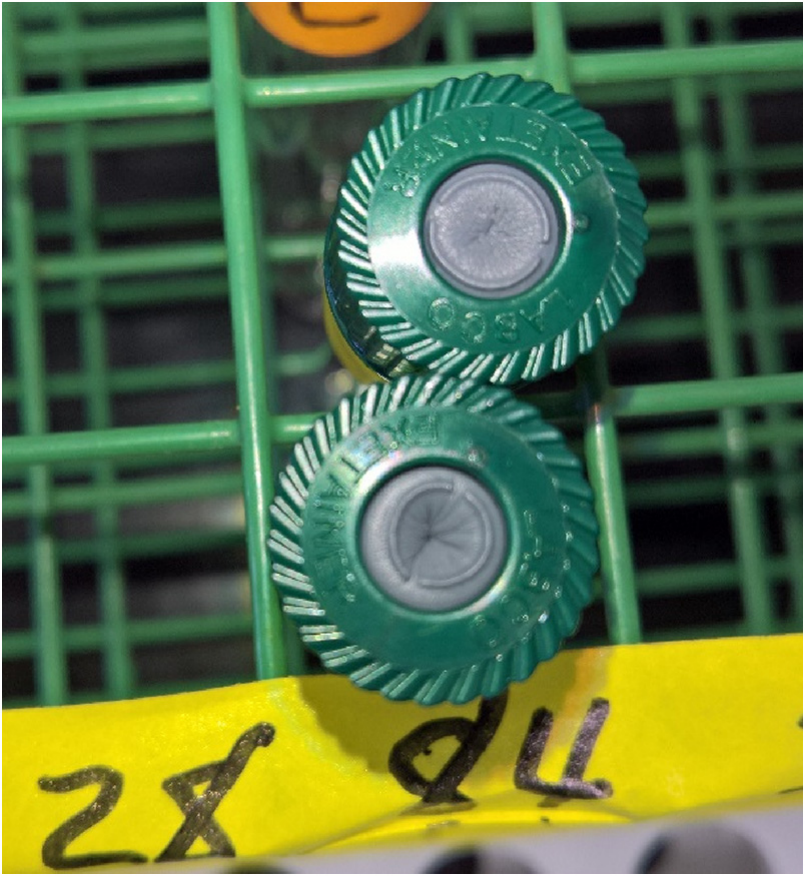
Photo showing denting in vial septa after 84 days of storage creating potential for sample leakage. Photo credit: Derek Faust.

However, CO_2_ concentrations less than certified in both standards after 84 days can be explained with literature regarding losses of N_2_O during storage. Possible explanations offered by Laughlin and Stevens [9] for greater N_2_O percent loss than for N_2_ included: a larger concentration gradient between Exetainers and the atmosphere; diffusion through septa; and adsorption to septum or glass. Since losses were observed for CO_2_, but not CH_4_ or N_2_O, the most likely explanation is leakage through or around septa because leakage rate from vials is proportional to the concentration gradient between the vials and ambient according to diffusion theory [5,9]. The certified CO_2_ concentration in the low standard (380 μL L^−1^) and high standard (1000 μL L^−1^) were at least one and two orders of magnitude greater, respectively, than the CH_4_ high (2.5 μL L^−1^) and N_2_O high (0.360 μL L^−1^) standard concentrations. Furthermore, loss of CO_2_ after 84 days of storage is analogous to increased probability of leaks at the collar/soil or collar/chamber interface with increasing deployment duration and soil gas diffusivity [1]. It is possible that leakage occurred around the septa of vials as a dent was observed in septa after 84 days, but not after any other storage time (Fig. 4). Although the severity of denting in septa was variable and not associated with any particular treatment, dents were observed in all septa after 84 days of storage, regardless of storage temperature.

## Conclusions

Based on results of this study, it is recommended that GHG samples in Exetainer vials with chlorobutyl septa be stored no longer than 28 days before analysis for CO_2_, CH_4_, and N_2_O. Though it may be possible to store samples up to or longer than 84 days, decreases in CO_2_ concentrations would be expected under similar conditions evaluated in this study. All storage temperatures evaluated in this study were found to be appropriate for storage of GHG samples. Accordingly, while a temperature-controlled environment with minimal light is ideal, a laboratory room with minimal temperature fluctuations is sufficient for GHG sample storage. These results provide scientifically supported guidance for storage of GHG samples. If the need arises for sample storage due to sample transport, analysis backlog, or inoperable instrumentation, then this study provides the scientific community confidence that samples can be properly stored for a period of 28 days without impacting data integrity. Furthermore, following guidance provided in this study will improve reliability of GHG values reported in the literature, ensure consistency, and help editors and reviewers assess data quality if samples were stored. Future studies should be conducted to further refine guidance on storage temperature and time for GHG samples.
